# Lung Cancer in the Course of COPD-Emerging Problems Today

**DOI:** 10.3390/cancers14153819

**Published:** 2022-08-06

**Authors:** Robert Uliński, Iwona Kwiecień, Joanna Domagała-Kulawik

**Affiliations:** 1 Doctoral School, Medical University of Warsaw, 02-091 Warsaw, Poland; 2 Laboratory of Hematology and Flow Cytometry, Department of Internal Medicine and Hematology, Military Institute of Medicine, 04-141 Warsaw, Poland; 3 Department of Internal Medicine, Pulmonary Diseases and Allergy, Medical University of Warsaw, 02-097 Warsaw, Poland

**Keywords:** COPD, lung cancer, tobacco smoking

## Abstract

**Simple Summary:**

Chronic obstructive pulmonary disease (COPD) is a risk factor for lung cancer independent of the influence of tobacco smoke. There are some similarities in immunopathogenesis of both diseases. The participation of inflammatory cells: macrophages, neutrophils and lymphocytes T with CD8 population was presented in both diseases. The emphysematous phenotype of COPD seems to be most often complicated by lung cancer. Some elements of autoimmunity could be involved in the development of emphysema, and its relation with malignant transformation requires further studies. COPD complicates lung cancer diagnosis and treatment, and the patients with coexistence of these both serious diseases need special care.

**Abstract:**

Tobacco smoking remains the main cause of tobacco-dependent diseases like lung cancer, chronic obstructive pulmonary disease (COPD), in addition to cardiovascular diseases and other cancers. Whilst the majority of smokers will not develop either COPD or lung cancer, they are closely related diseases, occurring as co-morbidities at a higher rate than if they were independently triggered by smoking. A patient with COPD has a four- to six-fold greater risk of developing lung cancer independent of smoking exposure, when compared to matched smokers with normal lung function. The 10 year risk is about 8.8% in the COPD group and only 2% in patients with normal lung function. COPD is not a uniform disorder: there are different phenotypes. One of them is manifested by the prevalence of emphysema and this is complicated by malignant processes most often. Here, we present and discuss the clinical problems of COPD in patients with lung cancer and against lung cancer in the course of COPD. There are common pathological pathways in both diseases. These are inflammation with participation of macrophages and neutrophils and proteases. It is known that anticancer immune regulation is distorted towards immunosuppression, while in COPD the elements of autoimmunity are described. Cytotoxic T cells, lymphocytes B and regulatory T cells with the important role of check point molecules are involved in both processes. A growing number of lung cancer patients are treated with immune check point inhibitors (ICIs), and it was found that COPD patients may have benefits from this treatment. Altogether, the data point to the necessity for deeper analysis and intensive research studies to limit the burden of these serious diseases by prevention and by elaboration of specific therapeutic options.

## 1. Introduction

### Epidemiology

Tobacco smoking remains a very common addiction in the population over 15 years old. It is the main cause of tobacco-dependent diseases, for example, lung cancer, chronic obstructive pulmonary disease (COPD), but also cardiovascular diseases and other cancers. Whilst the majority of smokers will not develop either COPD or lung cancer, they are closely related diseases, occurring as co-morbidities at a higher rate than if they were independently triggered by smoking. During the last few decades, smoking prevalence decreased from 26.7% in 2000 to 20.2% in 2015, and was projected to be 18.7% in 2020 globally [1,2]. Smoking is the most common in adults between 35–54 years old and more common among men 34.1% than among women 6.4% [1]. As the main group of smokers that smoked for decades will be soon in the age when COPD and lung cancer are the most common, the incidence of tobacco dependent diseases will still be increasing.

In 2020, lung cancer was surpassed by breast cancer, and it is the second most commonly diagnosed cancer, with 2.2 million new cases a year (11.4% of all cancers). Lung cancer remained the leading cause of cancer-related death, with an estimated 1.8 million deaths (18%) (Globocan 2020) [3]. In males, lung cancer is the leading cause of cancer morbidity, with 1.4 million cases (14.3%), and mortality (21.5%). In females, it remains the third most commonly diagnosed cancer, with 770 thousands cases (8.4%) after breast and colorectal cancer, and the second cause of cancer deaths (13.7%). Incidence and mortality rates are roughly two times higher in men than in women, but it varies worldwide with 1.2 times higher in North America and 5.6 in North Africa. Lung cancer is 3–4 times more common in developed countries than in developing countries, but this gap can change in the coming years, as 80% of tobacco smokers resided in low and middle income countries in 2016 [1]. Incidence and mortality in different countries reflect the extent of tobacco epidemics. It is the highest in North America and Europe and the lowest in Africa. Among women, the rates are also high in East Asia, which is caused by high outdoor ambient air pollution (PM2.5 air pollution was 20.5% in China in 2017) and exposure to household burning of solid fuels for heating and cooking [4,5]. In North America and several European countries, incidence rates among women are still increasing, approaching or equalling those among men, while among men they are stable or slightly decreasing, especially in younger generations [6,7]. The prognosis in lung cancer is very poor: only 10 to 20% of patients survive 5 years after diagnosis in most countries. However, the rates are higher in Japan (33%), and Israel (27%) [8].

COPD is the most common chronic pulmonary disease. According to the Global Burden of Disease Study 2017, all-age prevalent cases of COPD (i.e., individuals with COPD in 2017) were 300 million. Every year, 17.98 million new COPD cases are reported. The prevalence is the highest in high income countries, similar among men and women and has slightly increased since 1990 [9]. COPD is the third leading cause of death globally after ischemic heart disease and stroke, with an estimate of 3.324 million deaths (6% of all deaths in 2019) [10,11].

COPD is a risk factor for lung cancer development independent of smoking exposure with four- to six-fold greater risk of developing lung cancer, when compared to matched smokers with normal lung function. Every year, 0.8–1.7% of patients with COPD will be diagnosed with lung cancer, in comparison to only 0.2% risk in patients without COPD. The 10 year risk is about 8.8% in COPD group and only 2% in patients with normal lung function [12]. However, only 20% of smokers will develop COPD and 15% lung cancer, although they often die of other smoking-related causes like heart disease and stroke [13,14,15]. In about 50% of patients with COPD and lung cancer, COPD is recognised only during diagnostic procedures of lung tumour (supported by our observations). Lung cancer is also the most common cause of death (around 30% of cases) in patients with moderate COPD [16].

There is a question for clinicians—what to focus on, lung cancer in the course of COPD or to confirm COPD in patients with lung cancer? It seems most rational to have oncological vigilance in COPD and to include COPD in screening programs for lung cancer, whereas in patients with lung cancer, diagnostic of COPD is obligatory. The bibliography concerning the coexistence of both diseases is abundant, but sometimes concerning only selected problems [12]. Here, we present current data on lung cancer in COPD in many aspects.

## 2. Pathogenesis and Common Pathways

### 2.1. Inflammation

Pathogenesis of lung cancer and COPD share some common pathways. There are still many hypotheses of immunological, genetic and morphological similarities. The main common risk factors are tobacco smoking, exposure to household burning of solid fuels for heating and cooking, occupational exposure to dust and fumes, and living in polluted areas. Tobacco smoking is the most important and a very strong factor causing inflammation. Even after cessation of smoking, inflammation is still active [17,18]. Tobacco products inhaled with the air are accumulated in the airways and alveolar space. The mucosal surface is in direct contact with the external environment and is a major site of antigenic and toxic exposure in smokers. It has been reported that tobacco smoke contains over 4000 toxic chemicals, and of them, about 20 are carcinogens, as well as potent immuno-modulators, like lipopolysaccharide (LPS) [19]. Toxins including endotoxin associated with tobacco smoke may impact inflammation, as well as altering humoral- and cell-mediated immunity [20]. Tobacco-derived chemicals are absorbed by alveolar macrophages, which trigger an inflammatory chronic response in the lungs and increases over the years due to repeated exposure to smoke and the addition of new field-mutations across the pulmonary epithelia. Macrophages produce and release enzymes like proteases, which are involved in the inflammatory process of COPD and are responsible for the destruction of elastin fibres, fibronectin in the lung parenchyma and the beginning of emphysema, chemotactins and oxygen radicals [21]. There are three classes of proteases involved in COPD: serine, metalloproteinase and cysteine. In the group of serine proteases, neutrophil elastase, cathepsin G, and proteinase-3 are involved in the destruction of alveolar tissue. Matrix-metalloproteinase-9, 12, and 13 play an influential role in severity of COPD [22]. Among cysteine proteases, caspase-3, caspases-8 and caspase-9 play an important role in controlling apoptosis. The proteases activity can be regulated by inhibitors like α-1-antitrypsin, neutrophil elastase inhibitor, and leukocyte protease inhibitor. Studies suggest that neutrophil elastase may be a therapeutic target for COPD [21]. Alpha-1-antitrypsin deficiency can result in severe COPD in young patients [23].

Functional disorders in COPD: hyperinflation, airflow limitation and air trapping, as well as structural changes of the bronchial wall, cause decreased clearance of carcinogenetic substances, which leads to chronic inflammation, and predispose to the development of lung cancer [24]. The intrinsic pathway of carcinogenesis driven by classic mechanisms of carcinogen metabolic activation to DNA adducts is leading to miscoding and mutations in critical growth control genes, i.e., KRAS and P53. The extrinsic pathway driven by chronic inflammation present also in COPD started by macrophages with secretion of proteinases, cytokines, chemotaxins and oxygen radical species [25]. The macrophages number increases in smoking patients. In lung cancer, there is a special group of tumour associated macrophages (TAM), which are attracted by vascular endothelial growth factor (VEGF) and monocyte chemoatractant protein 1 (MCP-1). TAM consist of macrophages M1 (classically activated macrophages), which inhibit development of bacteria and lung cancer cells, and activate inflammation. M2 (alternatively activated macrophages) scavenge debris and promote angiogenesis, tissue remodelling, and repair. These macrophages are responsible for promotion of tumours and are more common in lung cancer tissue [25]. Lymphocytes T and B play an important role by maintaining inflammation.

Tobacco smoke increases the number of neutrophils in lung tissue. Their number increases significantly during COPD exacerbations. In lung cancer, there are two subpopulations of neutrophils N1 and N2, which are parts of TAN (tumour associated neutrophils). N1 and N2 neutrophils play opposite roles in progression of lung cancer and their ratio is an important factor [26].

Cigarette smoke contains around 10^15 oxygen radicals per one puff [27]. Additionally, inflammatory cells release oxygen radicals, which in COPD destruct lung tissue and in lung cancer they promote carcinogenesis, destructing DNA, which leads to miscoding and mutations in critical growth control genes.

Chronic inflammation in COPD is associated with the overexpression of the transcription factor NF-κB. It is a key mediator of carcinogenesis induced by inflammation, excessive proliferation and migration of immune cells [28]. NF-κB induces the expression of many pro-inflammatory cytokines such as interleukins: IL-1, IL-6, IL-8, tumour necrosis factor (TNF), the cyclins D1, D2, D3, E1 and various cyclin dependent kinases (CDKs) [28]. Moreover, NF-κB contribute to carcinogenesis by suppression of p53, by upregulating the levels of p53 E3 ligase, and thereby reducing p53 stability [12].

### 2.2. Immunity

**Figure 1:**
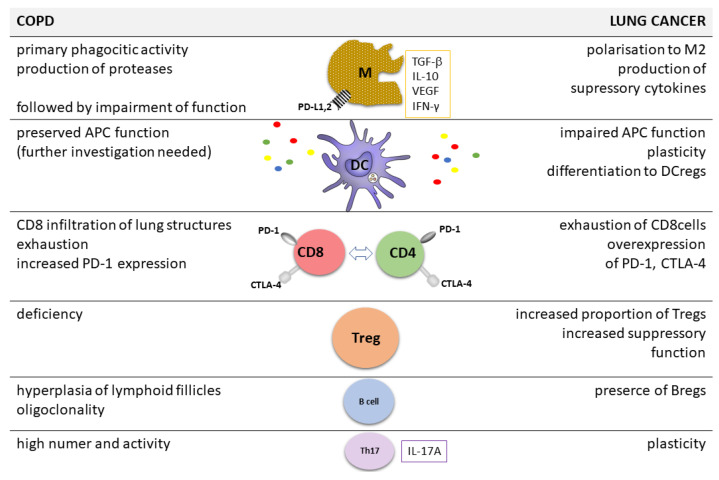
Main lymphoid cells involved in immune reaction in COPD and lung cancer. APC—antigen presenting cell, Bregreg—regulatory B cell, COPD—chronic obstructive pulmonary disease, CTLA-4—cytotoxic T cellll antigen-4, DC—dendritic cell, DC-reg—regulatory DC cell, IL—interleukin, IFN-γ—interferon γ, M—macrophage, PD-1—programmed death-1, TGF-β—transforming growth factor β. Treg-regulatory T cell, VEGF-vascular endothelial growth factor.

There is a growing body of evidence that immunological reactions not only protect organisms, but *per se* are involved in important pathological pathways. The impairment of immune anticancer response is responsible for tumour progression. These mechanisms are well known in lung cancer, which could be the best example of solid malignant tumour [29]. It was described in detail by plenty of original studies and reviews. Here, for illustration of the problem, we present only the main directions of complicated mechanisms of the escape of lung cancer from immune surveillance (Figure 1).

There are some immune pathways that overlap between the influence of tobacco smoke, COPD and lung cancer, while the others are different. Thus, the question arises—what is common, what immune mechanisms are responsible for supporting lung cancer development in the course of COPD? The role of immunity in the development of both diseases was supported by the study of Kachuri et al., in which the genetic background of a risk of lung cancer connected with airflow limitation concerned mainly immune-related pathways [30].

It should be taken into account that the common background in COPD and lung cancer exists—it is immune response distorted by tobacco smoking. Immune system of smokers is highly and permanently activated. The participation of inflammatory cells: neutrophils, macrophages, cytotoxic lymphocytes, and proinflammatory cytokines is known [31,32]. Immune and inflammatory processes are dynamic (response to chronic antigenic exposure nicely presented by Collier et al.) [33]. The direction of reactions evolves dependently on the local or systemic immune profile, the best example is plasticity of macrophages or transforming growth factor beta (TGFβ) [34,35]. The last being ubiquitous cytokine in smokers, COPD and lung cancer. Some kind of homeostasis in the airways and systemic environment is generated and persists long-term, even after smoking cessation [17]. However, when activated, the immune mechanisms become exhausted and many signs of impairment of immune system are observed. These are among others an extenuation of macrophages in their phagocytic function [36,37], exhaustion of cytotoxic lymphocytes [38], ineffective resolution of inflammation and clearance [39,40]. The immune process influenced by tobacco smoke is complicated by increased apoptosis of immune cells [41,42,43]. The products of apoptosis enhance inflammatory reactions and create a certain loop of injurious events. All of the above evolve from a simple defence reaction to tobacco smoke to pathogenesis of COPD and possible, carcinogenesis.

Tobacco smoke affects structural elements of the respiratory system. The well-known reactions of bronchial epithelium are squamous metaplasia to dysplasia and cancer in situ, gobbled cell metaplasia, non-specific inflammation and accumulation of B cells in lymphoid follicles [32,44]. These changes are numerous, but rather focal. The natural history of metaplasia is progression upon special circumstances, but in the majority the changes may heal and resolve. It could be assumed that the immune surveillance in COPD, which develops to that resembling lung cancer immunosuppression provides to progression of epithelial changes to early forms of carcinoma and further progression. The prevalence of squamous cell types of non-small cell lung cancer (NSCLC) in COPD confirms this pathogenetic course [45]. It was also found that tobacco smoke induced epithelial-to-mesenchymal transition and immune reaction specific only for COPD epithelium, not normal [46].

Two aspects of immune reaction in lung cancer in the course of COPD are worthy of elucidation. One is the role of lymphocytes, the second is autoimmunity as a suspected pathogenetic element.

#### 2.2.1. Lymphocytes

The participation of lymphocytes in COPD was documented in many studies [17,36,37,39,47,48,49]. These are mainly lymphocytes T CD8+. The augmentation of CD8 cells was observed in all layers of the bronchial wall, in the lung interstitium and circulation years ago [50]. Moreover, CD8 cells are found to be more specific for COPD than for “healthy” smokers and possible additional antigenic stimulus is responsible for CD8 recruitment [43]. Lymphocytes are involved in pathogenesis of COPD not only in the number, but also their function. One of the first works toward functional analysis of T cells was performed by Di Stefano et al., about 20 years ago [47]. They presented activated STAT-4+ cells in the airways of COPD patients and correlation with interferon γ (INFγ) concentration. A very important point presented in this study is the special balance of activation and suppression of T cells [47]. Recently, it is well-known that this balance is related to the activity of check point molecules, among others, by the programme death-1 (PD-1) suppressory molecule. The important role of PD-1-PD-L1 pathway in modulation of immune response in malignancy was well documented [51]. Other active molecules: suppressors and activators on T cells, were recognised [52,53]. There are much less data on the role of check point molecules in COPD than in lung cancer. In one of the studies, Wilkinson et al. suggested that expression of PD-1 on T cells is increased in COPD and leads to distorted anti-microbial defence [52]. In the excellent study by Biton et al., they analysed immune response in patients with coexisting COPD in lung cancer [38]. They confirmed the hypothesis that COPD alters the immune landscape in NSCLC. They analysed NSCLC tumour microenvironment (TME) by immunohistochemistry (IHC) and they found that in general COPD did not change the cellular profile in TME. However, the most affected by COPD was CD8 + T cell population with the number of PD-1 positive CD8 tumour infiltrating lymphocytes (TILs) correlated with the severity of COPD. The higher T cell exhaustion was observed in advanced COPD and CD8 population was mostly affected. The CD8 tumour high correlated with PD-L1 expression and was favourable for response to immune check point inhibitors (ICIs). Thus, COPD fits into a division of tumours to “hot” and “cold” ones [29], and seems to be “hot”, i.e., rich in immune cells infiltration, presence of PD-1/PD-L1 molecules and active cytokine network. Similar observations presented by Mark et al. [54] showed a better response to ICIs in the COPD group.

#### 2.2.2. Autoimmunity

There is evidence that, apart from the chronic inflammation caused by tobacco smoke, infections and recurrent exacerbations, the autoimmunity mechanisms are involved in the pathogenesis of COPD. The predisposition to autoimmune reaction in COPD may explain the occurrence of destructive changes forming new antigens and the participation of many immune alterations known to be active in autoreactivity. The metanalysis by Byrne et al. did not revealed uniform data on the participation of special autoantibodies in COPD [55]. It showed rather a lot of discrepancies in the results of the studies. Mainly anti-endothelial cell antibodies (AECA) and/or anti-epithelial cell autoantibodies showed association with COPD. The other autoantibodies in class IgM, IgA, IgG were identified individually. New antigens play an important role in pathogenesis of structural alterations in COPD. Neoantigens originate from own body structures and are recognised by the immune system. There are many causes of neoantigens formation in COPD. These are: cell damage, apoptosis, infections, products of proteases, foreign inhaled particles [48,49,56,57]. Apoptosis of structural elements of the respiratory system as well as of immune cells seems to play an important role in this autoimmune process in COPD. Destruction of the alveolar wall can create new antigens. Increase apoptosis of T cells was also presented. Protease activities enhance destruction in peripheral airways. The accumulation of the products of apoptosis cause reduced phagocytic capacities of macrophages and production of suppressory cytokines, among others TGFβ [37]. Thus, emphysema seems to be not only structural, but also immune disease [58,59].

Caramori et al. presented dynamic process leading to autoimmune mechanisms in COPD [60]. We show some modification in the Box 1.

Box 1 **Possible pathway of autoimmunity in COPD [60].**

              benign autoreactivity (great protection against autoimmunity in healthy lung)

                      pathological trigger (tobacco, pollutants)

                               ⇓

             disruption of immune protection → damage → new antigens → impaired clearance

                 new antigens + antibodies→ autoimmune reaction progression

The main cell populations connected with autoreactivity are recruited in COPD: B cells, CD8+ cells, Th17 cells, and regulatory T cells (Tregs). Tregs play a crucial role in the inhibition of the immune response. Their function depends on the expression of transcription factor forkhead box P3 (Foxp3) [61]. The presence of Cytotoxic T-lymphocyte antigen-4 (CTLA-4) molecule has been demonstrated as a strong inductor of Tregs function. Recently, some Tregs subpopulations were identified, and the stage of Tregs maturation was found to be important [62]. It was found that the population of Tregs is diminished in COPD [63,64,65].

The most active lymphocytes in autoreactivity are CD8+ cells, but T helper cells are needed in this cooperation. The prevalence of the Th1 profile of T cells was found in COPD. The supportive role of bystander cells in autoimmune reaction is suspected—in this context, T cells in different stages of maturation and some populations of natural killer T cells (NKT) are described [66]. Finally, the role of Th17 cells is well known in immune regulation. In COPD, expression of IL-17 is increased. It is associated with the severity of COPD and promotes chronic inflammation [67]. Interleukin-17A could become a new target for treatment of COPD-associated lung cancer. Lack of IL-17A, but not IL-17F, reduced tumour cell proliferation and inflammatory mediator expression [68]. Apart from lymphocytes, T and some B cell subpopulations are active in autoimmunity. These are among others: memory B cells, regulatory B cells (Bregs) and transitional B cells (trBcells) [57,69,70]. The pattern of the cytokine network in the context of cellular immune response is very important. The following cytokines are involved in autoimmune processes and regulation of immune response in the greatest extent: IL-10, TGFβ, IL-23, IL-17A. 

Some similar autoimmune mechanisms are reported in malignancy [57,71]. It is rational to suspect that a plethora of neoantigens and products of cell damage could activate autoreactivity [72]. However, the specific cellular and humoral elements of immune reaction in autoimmunity and cancer are at two poles [69]. Thus, this aspect of immune response in COPD/lung cancer patients is of great interest.

### 2.3. Genetic Candidates

Although COPD and lung cancer are nicotine-dependent diseases, only around 20% of smokers will develop COPD and 15% of them will develop lung cancer. It can be caused by genetic predisposition. The best known is the association between common variants in the *CHRNA5-CHRNA3-CHRNB4* nicotinic acetylcholine receptor subunit gene cluster on chromosome 15q25 and lung cancer risk [73]. These genes had been shown previously to be strongly associated with nicotine dependence [74] and lung cancer [75]. It was demonstrated that this association was likely due to smoking behaviour resulting in an increased uptake of nicotine and the lung carcinogen *NNK* in carriers of these variants [76]. There is also a significant body of evidence demonstrating that polymorphisms in CYP2A6 gene, the major gene for catalysis of nicotine metabolism, have a significant effect on smoking behaviour and possibly on lung cancer risk [77]. *FAM13A* is the second gene with a strong association with COPD and lung cancer. Additionally, it also predisposes to the development of asthma and pulmonary fibrosis. *FAM13A* gene encodes a protein, which presents tumour suppressor activity through inhibition of the intracellular signal transduction molecule RhoA [78]. Rho GTPases are also involved in the pulmonary endothelial barrier function in the lungs [79], which is often dysregulated in lung diseases, such as COPD and asthma. It was shown that genetic variants in *FAM13A* gene may determine susceptibility to COPD and lung cancer by dysregulating the repair processes in airways and leading to emphysema, fibrosis of small bronchi and chronic inflammation [78].

The Hedgehog (Hh)-interacting protein (HHIP) gene, is also associated with COPD and lung cancer [80]. HHIP is a regulator of the Hh signalling pathway, which has been shown to be vital for embryonic lung development and is also involved in mature airway epithelial repair [81,82]. Alterations of the HHIP protein or its expression may lead to changes in lung repair mechanisms, supporting a role in the development of COPD. In addition to this, the Hh signalling pathway is involved in EMT, mediating cigarette smoke-induced oncogenic transformation of bronchial epithelial cells and is necessary for cellular proliferation of many lung cancer cell lines [82,83].

### 2.4. Epigenetics

The new interesting pathway linking COPD and lung cancer are epigenetic changes of DNA. DNA hypomethylation is a ubiquitous feature of carcinogenesis. Cancer-linked hypomethylation occurs mostly in repeated DNA sequences, but gene regions show some too. DNA hypomethylation can be found early in carcinogenesis, but is also often associated with tumour progression [84]. Cigarette smoke has been demonstrated to impact global hypomethylation of repetitive genomic elements, and also methylation patterns of individual genes, which is relevant to nicotine addiction [85].

An epigenome wide association study (EWAS) identified in both: lung cancer and COPD patients a strong association with DNA methylation and repression of 2 genes, *CCDC37* and *MAP1B* [86]. Alterations to DNA methylation in COPD patients are mostly linked to hypomethylation of immune-modulatory genes or in the gene coding for alpha1-antitrypsin (SERPINA1 ) and linked to gene overexpression [87].

MicroRNAs (miRNA) are endogenous single-stranded RNAs that are 18–25 nucleotides in length. They can bind to full length mRNA and alter their translation into protein. The effect of inducing or repressing microRNA expression can influence many biological processes, including cell cycle, development, differentiation, proliferation, apoptosis, DNA repair, DNA methylation, and enhance or supress inflammation [88]. miR-1, miR-21 and mir-146a are linked with inflammation and proliferation [89,90]. Recently, Fathinavid et al. described possible miRNA examples involved in common pathways COPD and NSCLC, and these are: miR-106a, miR-17, miR-17, miR-15b, miR-107, and miR-103 [91]. 

## 3. Going to the Clinic

### 3.1. Comorbidity

Co-existence of COPD and lung cancer has very important clinical consequences and has strong influence on prognosis and treatment. The main risk of both diseases is tobacco smoking, but COPD is an independent risk factor of developing lung cancer. This predisposition supported by the knowledge on pathomechanisms indicates that a possible sequence is development of lung cancer in the course of COPD. Such an understanding of this co-morbidity may help in elaboration of clinical diagnostic–therapeutic strategies and in research studies.

Lung cancer is predominantly associated with older age than some cancers, i.e., breast cancer. The median age is around 70 years old, which is associated with the presence of comorbidities, which is the cause of treatment limitations, poorer outcome and quality of life. In one study, the majority of patients with lung cancer (87.3%) have at least one comorbidity: COPD (43%), renal impairment (28%) or ischaemic heart disease (27%) [92].

The most common comorbidities that have been associated with COPD include cardiovascular diseases, lung cancer, metabolic disorder, osteoporosis, anxiety and depression, skeletal muscle dysfunction, cachexia, gastrointestinal diseases, and other respiratory conditions [93]. Patients with COPD have mean 3.7 diagnosed diseases, compared with only 1.8 diseases in population [94]. It is very important to also optimally treat these diseases, because a lack of proper treatment of comorbid diseases decreases survival rate, increases exacerbations of COPD, and prolongs hospitalisation time [95]. Additionally, in patients with other pulmonary diseases, pulmonary symptoms can decrease oncologic vigilance of patients and physicians, which leads to delayed diagnosis of lung cancer. In patients with lung cancer, the most common pulmonary diseases obscuring clinical signs and symptoms are COPD, asthma and tuberculosis, but also pneumonia and silicosis [20].

COPD is not only a disease of the respiratory tract, but of the whole body. Chronic inflammation especially affects the cardiovascular system [96,97]. There is a significantly higher risk of coronary artery disease, angina and myocardial infarction [98], but also pulmonary hypertension, right heart hypertrophy [99], venous thromboembolism, pulmonary embolism [100] and acute ischemic stroke [101]. The systemic inflammatory response may cause endothelial injury and vascular dysfunction. Inflammatory cytokines produced by T cells and macrophages including IL-1, IL-6 and TNF-α all affect endothelial function [102], and increase coagulopathy. Plasma fibrin clots from patients with COPD are denser and more resistant to lysis [103]. Exacerbations of COPD with their associated neutrophil influx may be triggers for acute coronary events [96]. Palliative care of these patients is very important in the last years of life. In the last 6 months of life, patients with COPD are more likely to have a primary care visit and be admitted to an ICU, but less likely to receive palliative medications compared with patients with lung cancer. In patients with COPD and lung cancer together, the quality of life is worse [104].

### 3.2. COPD Is Not a Homogenic Disease

COPD is divided based on clinical characteristics into some distinct phenotypes. COPD phenotype refers to a single or combination of disease attributes that describe differences between COPD patients based on clinically significant parameters, such as exacerbation, symptoms, response to treatment, rate of disease progression, and mortality (qualified as A,B,C,D groups) [16]. Old classification of phenotypes were A (patients with chronic bronchitis, inflammatory phenotype, frequent exacerbator, systemic manifestations and with co-morbidities) and B (patients with emphysema, pronounced lung hyperinflation and without frequent exacerbations). However, the reality is that many more phenotypes are likely to exist [105,106,107].

Widely accepted COPD phenotypes are presented in Table 1.

From this complex clinical picture of COPD a question arises—in which one is the risk of lung cancer highest? A phenotype with predominance of emphysema was described as mostly affected by malignancy [124,125], and the prevalence of centrilobular emphysema was described [111]. It could be explained by structural changes of lung parenchyma and by a character of immune alterations with direction to those observed in lung cancer. It could be suspected that in phenotypes with chronic bronchitis, the persistent activation of immune system and inflammation maintains a protective character against malignant transformation.

### 3.3. Difficulties in Diagnosis

A basis of lung cancer diagnosis is histological confirmation, for COPD: clinical signs, risk factors and results of spirometry [16]. As mentioned above the symptoms and signs of both diseases are similar and often overlapping. Some of them may be ignored by patients as typical for tobacco smoking. Thus, the knowledge on coexistence of both diseases is necessary to perform a proper diagnostic plan and to take it into consideration. A good practice is planning for spirometry during diagnosis of lung cancer. On the other hand, the COPD patient needs careful medical care with additional examinations such as chest X-rays or even chest computed tomography (CT). It was presented that COPD should be included in the qualification of lung cancer screening. The usefulness of COPD–lung cancer screening score–diffusing capacity for carbon monoxide (COPD-LUCSS- DLCO) in early lung cancer diagnosis was recently documented and are discussed by Sousa et al. [126]. The incorporation of emphysema detected in low dose CT, spirometry or DLCO in addition to age and smoking history to screening programs importantly improved identification of patients with lung cancer risk [126,127]. Unfortunately, COPD is often a contraindication to diagnostic procedures: bronchoscopy, transthoracic needle aspiration, and cryobiopsy. The risk of respiratory failure and pneumothorax in patients with COPD is much higher than in patients with normal lung function and without emphysema.

### 3.4. COPD Influence Treatment of Lung Cancer

The most powerful therapeutic approach for NSCLC is lung surgical resection. This treatment is possible mainly in stage I, II and IIIa [128]. However, this option is associated with higher morbidity and mortality in patients with low ventilatory reserve, which is a common limiting factor for lung cancer surgery in patients with COPD [129,130]. In this way, preoperative risk assessment should be performed to achieve an accurate risk stratification. Many parameters have been proposed as predictors of perioperative risk. They include functional parameters at rest: forced expiratory volume in the first second (FEV1) and DLCO, peak VO2 (maximal oxygen uptake), predicted postoperative FEV1 (ppoFEV1), ppoDLCO and ppoVO2 values. In patients with FEV1 and DLCO more than 80% of predicted, it is safe to perform lung resection up to pneumonectomy. If any of these values are less than 80%, further investigations should be performed as exercise testing and peak V02. Pneumonectomy is safe to perform if VO2 is more than 20 mL/kg/min. If it is less than 10 mL/kg/min pneumonectomy or lobectomy is usually not recommended. If V02 is between 10 and 20 mL/kg/min split function ppoFEV1 and ppoDLCO should be calculated. Both should be above 30% to perform surgery up to the calculated extent. If any is less than 30%, we should calculate ppoV02. PpoV02 less than 10 mL/kg/min will disqualify the patient from pneumonectomy or lobectomy [131].

Many case series in the past have shown that peri-operative risks increase substantially when ppo-FEV1 is 40% of predicted, reporting mortality rates ranging 16–50% [132,133]. More recently, in those patients with a ppo-FEV1 40%, the mortality rate was only 4.8% [134]. These findings have been partly explained by the so-called ‘‘lung volume reduction effect’’ that can reduce the functional loss in patients with airflow limitations. In this regard, many studies have already shown the minimal loss, or even improvement, in pulmonary function after lobectomy in lung cancer patients with moderate to severe COPD, questioning the traditional operability criteria mostly based on pulmonary parameters [135,136,137]. A value for ppo-FEV1 of 30% is currently used to distinguish between normal risk and higher risk lung resection patients. However, the ppo-FEV1 should not be used alone to select patients with lung cancer for lung resection, particularly patients with moderate to severe COPD.

Patients with primary lung cancer and emphysema can be ideal candidates for lung volume resection surgery with concomitant resection of tumours. Patients need to have moderately impaired FEV1 between 30% and 65% and evidence of emphysema in DLCO or CT. We should consider mass localisation and anatomy of emphysema to choose proper patients for this strategy [96].

For patients with stage I peripheral lung cancer with contraindication to surgery the stereotactic radiotherapy (SABR or SBRT) is recommended [128]. This therapy is safe for patients with COPD and the elderly.

The main problem of lung cancer is the great proportion of advanced stages at recognition. There is about 60% of patients beyond the possibility of radical treatment. Different kinds of systemic therapy are used in this group of patients [138]. To date, no special systemic regimen is recommended for patients with coexistent COPD. However, it is promising that having active anticancer response, rich immune network with CD8 cells and high PD-1-PD-L1 axis and, probably, tumour mutational burden (TMB) patients with COPD could have benefit from immunotherapy with ICIs. However, TMB was not validated as a marker in predicting response to immunotherapy to date. Of course, a new problem opens—this group may be affected by immune-related adverse events (irAE) [139,140]. It is the next problem for observational studies.

## 4. Conclusions

COPD and lung cancer form, each and together, a global problem not only in developed countries. Tobacco smoking is still the main causative agent for both diseases. The incidence of both diseases increases in both sexes in spite of the tendency to decrease tobacco smoking. COPD was found to be a risk factor for lung cancer independently of the influence of tobacco smoke. It is the reason for the necessity to advise special caution to this group of patients. COPD phenotype with predominance of emphysema seems to form structural changes, which can lead to malignant transformation. It is postulated to include the signs of COPD: detection of emphysema in chest imaging and the results of pulmonary function test into the screening program for lung cancer to improve detection of tumours in early stages. However, COPD importantly limits the possibility of radical surgical treatment of lung cancer in the first stages. The new therapeutic modalities are promising in this group of patients. Immunotherapy with immune check point inhibitors showed effectiveness that may be explained by special immune constellation resembling “hot” tumours. The character of immune reaction and chronic inflammation in patients with lung cancer and COPD was partly recognised and need further studies.

## Figures and Tables

**Table 1:** Characteristic of phenotypes of chronic obstructive pulmonary disease (COPD) and relation with lung cancer.

Phenotype	Characteristic	Reference
Chronic bronchitis	Associated with an accelerated lung function decline and an increased risk of respiratory infections, macrolide antibiotics with anti-inflammatory properties and phosphodiesterase- 4 inhibitors have been used to decrease COPD exacerbations and may be beneficial in the treatment.	[108,109,110]
Emphysematous	Presence of emphysema confirmed on chest imaging, relative to non-emphysema-predominant phenotype of COPD, emphysema-predominant phenotype has a higher risk of squamous-cell carcinoma and small-cell lung cancer, especially the centrilobular phenotype of emphysema increase lung cancer risk.	[111,112]
Asthma-COPD-Overlap (ACO)	Persistent airflow limitation usually with increased variability in airflow, airway obstruction is usually incompletely reversible and patients have several features associated with asthma as eosinophilia in sputum, personal history of asthma, high total IgE, personal history of atopy, and inhaled corticosteroids is an important treatment.	[113,114]
Frequent exacerbator	Presence of frequent exacerbations of two or more per year, there is three-fold increase of mortality in this phenotype.	[115]
**Emerging COPD phenotypes**		
Pulmonary cachexia phenotype	With BMI lower than 21 kg/m^2^, extra-pulmonary degenerative manifestations include osteoporosis and muscle wasting (relatively high in COPD: 15–40% depending on definition and disease stage), muscle wasting not only contributes to diminished skeletal muscle function, reduced exercise capacity, but is also a determinant of mortality in COPD, independent of airflow obstruction.	[116,117,118]
Physical frailty phonotype	With weakness, slowness, low-level of physical activity, self-reported exhaustion and unintentional loss of weight, worse airflow limitation and symptoms, pulmonary rehabilitation can be effective.	[119]
Emotional frailty phenotype	Characterise by anxiety, depression, fear of breathlessness and is associated with increased morbidity, mortality and hospitalisation, patients should be supported by cognitive behavioural therapy.	[120]
Overlap COPD and bronchiectases	Confirmation of bronchiectases in HRCT and definite COPD diagnosis, both diseases have similar symptoms, because of that proper diagnosis and differentiation is a challenge, treatment useful in COPD may not be widely effective in bronchiectasis and vice versa (like ICS).	[121]
Upper lobe-predominant emphysema	Diagnosed by CT findings consistent of predominant upper lobe emphysema, significant improvement after LVRS.	[122]
Fast decliner phenotype	With rapid decline of lung function and high mortality, these patients should be early referred to specialised centres for aggressive disease management, lung transplantation should be considered early.	[123]
The comorbidities or systemic phenotype	High comorbidities burden, predominantly cardiovascular and metabolic, patients have increased mortality risk and should be managed by multispecialist teams.	[123]
α1-antitrypsin deficiency (AATD)	Genetic disorder associated with early onset COPD, highly under-diagnosed condition, early diagnosis could prompt specific interventions such as smoking cessation, testing of family members, genetic counselling and use of replacement therapy.	[23]
No smoking COPD	Induced by biomass exposure, especially among women in East Asia, which is caused by high outdoor ambient air pollution and exposure to household burning of solid fuels for heating and cooking.	[4,5]

BMI—Body Mass Index, HRCT—high resolution computed tomography, ICS—inhaled corticosteroids, LVRS—lung volume reduction surgery.

## Data Availability

Not applicable.
